# Tris{2-[(pyrimidin-2-yl-κ*N*)amino­meth­yl]phenol}silver(I) nitrate

**DOI:** 10.1107/S1600536812046119

**Published:** 2012-11-28

**Authors:** Shan Gao, Seik Weng Ng

**Affiliations:** a Key Laboratory of Functional Inorganic Material Chemistry, Ministry of Education, Heilongjiang University, Harbin 150080, People’s Republic of China; b Department of Chemistry, University of Malaya, 50603 Kuala Lumpur, Malaysia; c Chemistry Department, Faculty of Science, King Abdulaziz University, PO Box 80203 Jeddah, Saudi Arabia

## Abstract

The Ag^I^ atom in the title compound, [Ag(C_11_H_11_N_3_O)_3_]NO_3_, shows a *T*-shaped coordination arising from bonding to the N atom of three N-heterocycles; the geometry is distorted towards square pyramidal owing to two weak Ag⋯O_nitrate_ inter­actions [Ag⋯O = 2.691 (5) and 3.073 (5) Å]. The cation and anion are linked by O—H⋯N and N—H⋯O hydrogen bonds, generating a three-dimensional network.

## Related literature

For the structure of the 2-{[(pyrimidin-2-yl)amino]­meth­yl}phenol ligand, see: Xu *et al.* (2011 ▶).
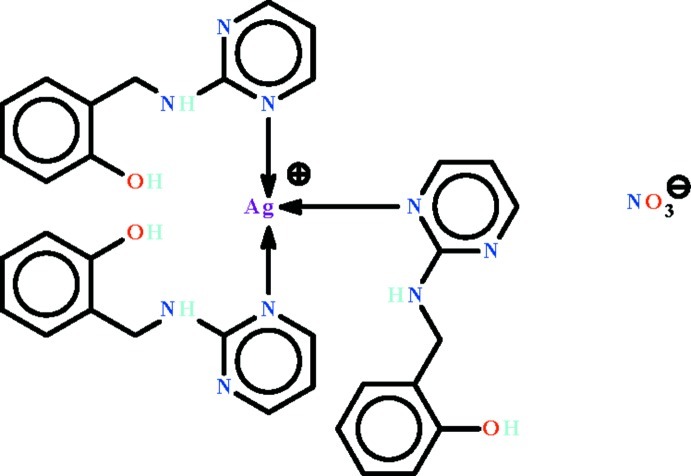


## Experimental

### 

#### Crystal data

[Ag(C_11_H_11_N_3_O)_3_]NO_3_
*M* *_r_* = 773.56
Triclinic, 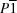

*a* = 7.5987 (4) Å
*b* = 13.7931 (7) Å
*c* = 16.1308 (10) Å
α = 89.159 (2)°
β = 88.236 (2)°
γ = 82.777 (1)°
*V* = 1676.35 (16) Å^3^
*Z* = 2
Mo *K*α radiation
μ = 0.66 mm^−1^
*T* = 293 K
0.25 × 0.20 × 0.15 mm

#### Data collection

Rigaku R-AXIS RAPID IP diffractometer
Absorption correction: multi-scan (*ABSCOR*; Higashi, 1995 ▶) *T*_min_ = 0.852, *T*_max_ = 0.907
16545 measured reflections
7609 independent reflections
3959 reflections with *I* > 2σ(*I*)
*R*_int_ = 0.065

#### Refinement

*R*[*F* ^2^ > 2σ(*F* ^2^)] = 0.062
*wR*(*F* ^2^) = 0.177
*S* = 1.05
7609 reflections
470 parameters
6 restraints
H atoms treated by a mixture of independent and constrained refinement
Δρ_max_ = 1.17 e Å^−3^
Δρ_min_ = −1.20 e Å^−3^

### 

Data collection: *RAPID-AUTO* (Rigaku, 1998 ▶); cell refinement: *RAPID-AUTO*; data reduction: *CrystalClear* (Rigaku/MSC, 2002 ▶); program(s) used to solve structure: *SHELXS97* (Sheldrick, 2008 ▶); program(s) used to refine structure: *SHELXL97* (Sheldrick, 2008 ▶); molecular graphics: *X-SEED* (Barbour, 2001 ▶); software used to prepare material for publication: *publCIF* (Westrip, 2010 ▶).

## Supplementary Material

Crystal structure: contains datablock(s) global, I. DOI: 10.1107/S1600536812046119/xu5645sup1.cif

Structure factors: contains datablock(s) I. DOI: 10.1107/S1600536812046119/xu5645Isup2.hkl

Additional supplementary materials:  crystallographic information; 3D view; checkCIF report

## Figures and Tables

**Table 1:** Hydrogen-bond geometry (Å, °)

*D*—H⋯*A*	*D*—H	H⋯*A*	*D*⋯*A*	*D*—H⋯*A*
O1—H1o⋯N8^i^	0.84 (1)	2.00 (3)	2.800 (7)	158 (9)
O2—H2o⋯N5^ii^	0.84 (1)	1.95 (1)	2.788 (6)	177 (9)
O3—H3o⋯N2^iii^	0.84 (1)	1.99 (2)	2.818 (6)	171 (8)
N3—H3n⋯O4	0.88 (1)	2.22 (2)	3.073 (7)	164 (6)
N6—H6n⋯O5^iv^	0.88 (1)	2.09 (4)	2.879 (8)	149 (6)
N9—H9n⋯O6^iv^	0.88 (1)	2.28 (4)	3.033 (8)	143 (6)

Symmetry codes: (i) 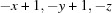; (ii) 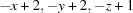; (iii) 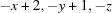; (iv) 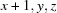.

**Fig. 1.**
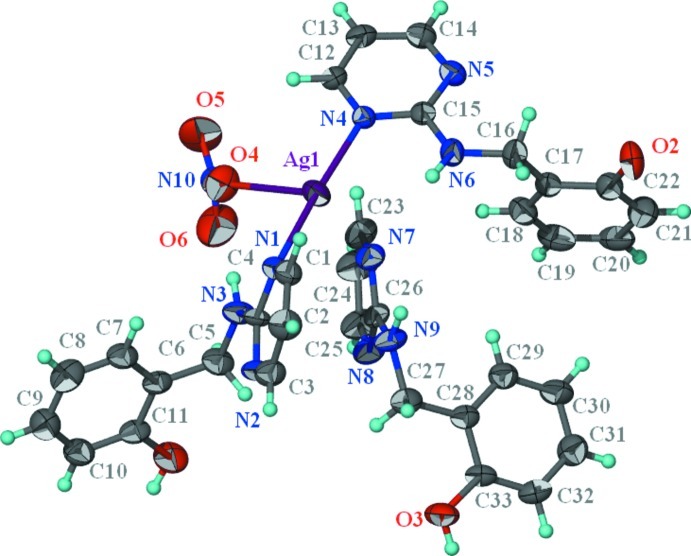
Thermal ellipsoid plot (Barbour, 2001) of [Ag(C11H11N3O)3] NO3 at the 50% probability level; hydrogen atoms are drawn as spheres of arbitrary radius. The weak Ag···O interactions are shown as dashed lines.

## supplementary crystallographic information

### Comment

A recent study reports 2-[(pyrimidin-2-ylamino)methyl]phenol, a reduced Schiff-base that possesses an acidic phenolic group (Xu *et al.*, 2011). The reaction with silver nitrate yields the salt, [Ag(C_11_H_11_N_3_O)_3_] NO_3_, in which the metal center shows *T*-shaped coordination arising from bonding to the N atom of three *N*-heterocycles (Scheme I, Fig. 1). The geometry is distorted towards a square pyramidal owing to two Ag···O_nitrate_ interactions. The same O atom of the nitrate is involved, *i.e.*, this atom engages in weak bridging to generate a dinuclear cation [Ag—O 2.691 (5), 3.073 (5) Å]. The cation and anion are linked by O–H···N and N–H···O hydrogen bonds to generate a three-dimensional network (Table 1).

### Experimental

An acetonitrile solution (10 ml) of silver nitrate (1 mmol) was added to a methanol solution (5 ml) of 2-[(pyriamidin-2-ylamino)methyl]phenol (1 mmol) and aqueous ammonium hydroxide (0.5 mmol). The solution was filtered and then side aside, away from light, for the growth of crystals. Colorless crystals were obtained after several days.

### Refinement

Hydrogen atoms were placed in calculated positions (C–H 0.93–0.97 Å) and were included in the refinement in the riding model approximation, with *U*(H) set to 1.2*U*(C). The amino and hydroxy H atoms were located in a difference Fouier map, and were refined with distance restraints of N–H 0.88±0.01 and O–H 0.84±0.01 Å. Their temperature factors were refined tied by a factor of 1.5 times for O and 1.2 times for N.

The final difference Fourier map had a peak at 1.40 Å from N1 and a hole at 0.95 Å from Ag1.

### Crystal data

**Table:**

[Ag(C_11_H_11_N_3_O)_3_]NO_3_	*Z* = 2
*M**_r_* = 773.56	*F*(000) = 792
Triclinic, *P*1	*D*_x_ = 1.533 Mg m^−^^3^
Hall symbol: -P 1	Mo *K*α radiation, λ = 0.71073 Å
*a* = 7.5987 (4) Å	Cell parameters from 8487 reflections
*b* = 13.7931 (7) Å	θ = 3.0–27.5°
*c* = 16.1308 (10) Å	µ = 0.66 mm^−^^1^
α = 89.159 (2)°	*T* = 293 K
β = 88.236 (2)°	Prism, colorless
γ = 82.777 (1)°	0.25 × 0.20 × 0.15 mm
*V* = 1676.35 (16) Å^3^	

### Data collection

**Table:**

Rigaku R-AXIS RAPID IP diffractometer	7609 independent reflections
Radiation source: fine-focus sealed tube	3959 reflections with *I* > 2σ(*I*)
Graphite monochromator	*R*_int_ = 0.065
ω scan	θ_max_ = 27.5°, θ_min_ = 3.0°
Absorption correction: multi-scan (*ABSCOR*; Higashi, 1995)	*h* = −9→9
*T*_min_ = 0.852, *T*_max_ = 0.907	*k* = −17→16
16545 measured reflections	*l* = −20→20

### Refinement

**Table:**

Refinement on *F*^2^	Secondary atom site location: difference Fourier map
Least-squares matrix: full	Hydrogen site location: inferred from neighbouring sites
*R*[*F*^2^ > 2σ(*F*^2^)] = 0.062	H atoms treated by a mixture of independent and constrained refinement
*wR*(*F*^2^) = 0.177	*w* = 1/[σ^2^(*F*_o_^2^) + (0.0548*P*)^2^ + 4.1203*P*] where *P* = (*F*_o_^2^ + 2*F*_c_^2^)/3
*S* = 1.05	(Δ/σ)_max_ = 0.001
7609 reflections	Δρ_max_ = 1.17 e Å^−^^3^
470 parameters	Δρ_min_ = −1.20 e Å^−^^3^
6 restraints	Extinction correction: *SHELXL97* (Sheldrick, 2008), Fc^*^=kFc[1+0.001xFc^2^λ^3^/sin(2θ)]^-1/4^
Primary atom site location: structure-invariant direct methods	Extinction coefficient: 0.0050 (9)

### Fractional atomic coordinates and isotropic or equivalent isotropic displacement parameters (Å^2^)

**Table:**

	*x*	*y*	*z*	*U*_iso_*/*U*_eq_	
Ag1	0.69762 (7)	0.56100 (4)	0.41785 (3)	0.0542 (2)	
O1	0.3868 (7)	0.3857 (4)	0.0184 (3)	0.0694 (14)	
H1O	0.357 (11)	0.362 (6)	−0.026 (3)	0.104*	
O2	1.2106 (7)	0.9698 (3)	0.4381 (3)	0.0667 (14)	
H2O	1.249 (11)	1.023 (3)	0.447 (5)	0.100*	
O3	1.1119 (7)	0.6138 (4)	−0.0349 (3)	0.0638 (13)	
H3O	1.132 (11)	0.623 (6)	−0.0857 (13)	0.096*	
O4	0.3660 (6)	0.5147 (4)	0.4050 (3)	0.0706 (14)	
O5	0.1076 (7)	0.5819 (5)	0.4368 (4)	0.102 (2)	
O6	0.1982 (9)	0.5706 (6)	0.3104 (4)	0.113 (2)	
N1	0.8040 (6)	0.4319 (3)	0.3438 (3)	0.0402 (11)	
N2	0.8554 (6)	0.3658 (4)	0.2085 (3)	0.0461 (13)	
N3	0.5836 (6)	0.4482 (4)	0.2477 (3)	0.0514 (14)	
H3N	0.505 (6)	0.472 (4)	0.286 (3)	0.062*	
N4	0.6185 (6)	0.6924 (3)	0.4963 (3)	0.0395 (11)	
N5	0.6519 (7)	0.8562 (4)	0.5321 (3)	0.0479 (13)	
N6	0.8682 (7)	0.7597 (4)	0.4565 (3)	0.0494 (13)	
H6N	0.911 (8)	0.702 (2)	0.437 (4)	0.059*	
N7	0.6476 (7)	0.6835 (4)	0.2894 (3)	0.0527 (14)	
N8	0.6182 (7)	0.6870 (4)	0.1425 (3)	0.0568 (15)	
N9	0.8789 (7)	0.6212 (4)	0.2025 (3)	0.0535 (14)	
H9N	0.934 (8)	0.620 (5)	0.250 (2)	0.064*	
N10	0.2226 (7)	0.5580 (4)	0.3840 (4)	0.0500 (13)	
C1	0.9638 (8)	0.3903 (4)	0.3636 (4)	0.0480 (15)	
H1A	1.0024	0.3994	0.4167	0.058*	
C2	1.0762 (9)	0.3342 (5)	0.3094 (5)	0.0600 (18)	
H2A	1.1862	0.3033	0.3252	0.072*	
C3	1.0165 (8)	0.3265 (5)	0.2311 (4)	0.0535 (17)	
H3A	1.0913	0.2925	0.1918	0.064*	
C4	0.7503 (7)	0.4140 (4)	0.2663 (3)	0.0388 (14)	
C5	0.5075 (8)	0.4352 (5)	0.1674 (4)	0.0536 (17)	
H5A	0.5903	0.4518	0.1241	0.064*	
H5B	0.3997	0.4805	0.1628	0.064*	
C6	0.4642 (7)	0.3328 (5)	0.1527 (4)	0.0441 (15)	
C7	0.4823 (8)	0.2587 (5)	0.2110 (4)	0.0523 (17)	
H7	0.5260	0.2707	0.2625	0.063*	
C8	0.4378 (9)	0.1679 (6)	0.1953 (5)	0.064 (2)	
H8	0.4509	0.1195	0.2362	0.077*	
C9	0.3732 (10)	0.1475 (6)	0.1188 (5)	0.066 (2)	
H9	0.3426	0.0857	0.1082	0.079*	
C10	0.3548 (9)	0.2194 (5)	0.0590 (4)	0.0579 (18)	
H10	0.3120	0.2065	0.0075	0.069*	
C11	0.4003 (8)	0.3117 (5)	0.0754 (4)	0.0471 (15)	
C12	0.4615 (8)	0.7019 (5)	0.5363 (4)	0.0453 (15)	
H12	0.3968	0.6489	0.5386	0.054*	
C13	0.3924 (8)	0.7870 (5)	0.5742 (4)	0.0512 (16)	
H13	0.2818	0.7933	0.6013	0.061*	
C14	0.4937 (8)	0.8625 (5)	0.5701 (4)	0.0503 (16)	
H14	0.4492	0.9211	0.5953	0.060*	
C15	0.7105 (7)	0.7705 (4)	0.4960 (3)	0.0387 (13)	
C16	0.9941 (8)	0.8302 (4)	0.4533 (4)	0.0486 (16)	
H16A	1.1123	0.7955	0.4449	0.058*	
H16B	0.9905	0.8623	0.5065	0.058*	
C17	0.9631 (8)	0.9068 (4)	0.3869 (4)	0.0439 (14)	
C18	0.8294 (9)	0.9114 (5)	0.3308 (4)	0.0556 (17)	
H18	0.7534	0.8635	0.3325	0.067*	
C19	0.8050 (11)	0.9855 (6)	0.2719 (4)	0.067 (2)	
H19	0.7127	0.9884	0.2350	0.080*	
C20	0.9214 (12)	1.0555 (5)	0.2690 (5)	0.071 (2)	
H20	0.9086	1.1046	0.2287	0.085*	
C21	1.0550 (11)	1.0534 (5)	0.3244 (5)	0.068 (2)	
H21	1.1292	1.1021	0.3231	0.082*	
C22	1.0783 (9)	0.9791 (5)	0.3818 (4)	0.0522 (16)	
C23	0.4882 (10)	0.7330 (6)	0.2965 (5)	0.065 (2)	
H23	0.4410	0.7468	0.3495	0.078*	
C24	0.3878 (10)	0.7654 (7)	0.2303 (5)	0.080 (2)	
H24	0.2793	0.8045	0.2367	0.096*	
C25	0.4575 (10)	0.7367 (7)	0.1543 (5)	0.074 (2)	
H25	0.3890	0.7526	0.1081	0.088*	
C26	0.7118 (9)	0.6650 (5)	0.2116 (4)	0.0468 (15)	
C27	0.9738 (9)	0.6094 (5)	0.1229 (4)	0.0555 (17)	
H27A	0.9018	0.5787	0.0850	0.067*	
H27B	1.0826	0.5656	0.1303	0.067*	
C28	1.0201 (7)	0.7037 (5)	0.0839 (4)	0.0435 (14)	
C29	0.9935 (8)	0.7930 (5)	0.1241 (4)	0.0518 (16)	
H29	0.9406	0.7967	0.1769	0.062*	
C30	1.0442 (9)	0.8758 (5)	0.0871 (4)	0.0636 (19)	
H30	1.0289	0.9344	0.1158	0.076*	
C31	1.1173 (9)	0.8728 (5)	0.0081 (5)	0.0623 (19)	
H31	1.1494	0.9293	−0.0170	0.075*	
C32	1.1429 (8)	0.7854 (5)	−0.0336 (4)	0.0534 (17)	
H32	1.1945	0.7827	−0.0867	0.064*	
C33	1.0917 (8)	0.7016 (5)	0.0034 (4)	0.0450 (15)	

### Atomic displacement parameters (Å^2^)

**Table:**

	*U*^11^	*U*^22^	*U*^33^	*U*^12^	*U*^13^	*U*^23^
Ag1	0.0598 (3)	0.0507 (3)	0.0529 (4)	−0.0096 (2)	0.0024 (2)	−0.0169 (2)
O1	0.088 (4)	0.079 (4)	0.044 (3)	−0.015 (3)	−0.024 (3)	0.006 (3)
O2	0.065 (3)	0.049 (3)	0.090 (4)	−0.022 (2)	−0.015 (3)	0.003 (3)
O3	0.089 (3)	0.061 (3)	0.040 (3)	−0.004 (3)	0.009 (3)	−0.010 (2)
O4	0.043 (3)	0.082 (4)	0.086 (4)	−0.003 (3)	−0.008 (3)	0.007 (3)
O5	0.075 (4)	0.101 (5)	0.124 (5)	0.011 (3)	0.042 (4)	−0.045 (4)
O6	0.121 (5)	0.152 (6)	0.073 (5)	−0.046 (5)	−0.036 (4)	0.039 (4)
N1	0.040 (3)	0.044 (3)	0.036 (3)	−0.003 (2)	0.000 (2)	−0.007 (2)
N2	0.044 (3)	0.063 (3)	0.031 (3)	−0.005 (3)	0.006 (2)	−0.011 (2)
N3	0.038 (3)	0.072 (4)	0.042 (3)	0.002 (3)	0.002 (2)	−0.017 (3)
N4	0.051 (3)	0.034 (3)	0.034 (3)	−0.009 (2)	0.002 (2)	−0.001 (2)
N5	0.056 (3)	0.040 (3)	0.047 (3)	−0.005 (2)	0.003 (3)	−0.009 (2)
N6	0.051 (3)	0.038 (3)	0.060 (4)	−0.010 (3)	0.012 (3)	−0.007 (3)
N7	0.054 (3)	0.072 (4)	0.035 (3)	−0.017 (3)	−0.006 (2)	0.008 (3)
N8	0.052 (3)	0.084 (4)	0.036 (3)	−0.013 (3)	−0.008 (3)	0.010 (3)
N9	0.052 (3)	0.079 (4)	0.029 (3)	−0.006 (3)	0.001 (2)	0.005 (3)
N10	0.047 (3)	0.050 (3)	0.054 (4)	−0.008 (3)	0.002 (3)	−0.005 (3)
C1	0.056 (4)	0.048 (4)	0.041 (4)	−0.009 (3)	−0.003 (3)	−0.002 (3)
C2	0.046 (4)	0.070 (5)	0.062 (5)	0.002 (3)	−0.004 (3)	−0.004 (4)
C3	0.044 (4)	0.064 (4)	0.051 (4)	−0.002 (3)	0.004 (3)	−0.006 (3)
C4	0.041 (3)	0.052 (4)	0.024 (3)	−0.004 (3)	0.001 (2)	−0.012 (3)
C5	0.045 (3)	0.070 (5)	0.046 (4)	−0.003 (3)	−0.011 (3)	−0.008 (3)
C6	0.034 (3)	0.064 (4)	0.033 (3)	−0.003 (3)	−0.002 (2)	−0.001 (3)
C7	0.046 (4)	0.073 (5)	0.038 (4)	−0.008 (3)	−0.008 (3)	−0.005 (3)
C8	0.058 (4)	0.075 (5)	0.059 (5)	−0.002 (4)	−0.007 (4)	0.020 (4)
C9	0.070 (5)	0.062 (5)	0.067 (5)	−0.012 (4)	0.001 (4)	−0.005 (4)
C10	0.059 (4)	0.072 (5)	0.045 (4)	−0.012 (4)	−0.008 (3)	−0.006 (4)
C11	0.047 (3)	0.064 (4)	0.029 (3)	−0.002 (3)	−0.008 (3)	0.006 (3)
C12	0.051 (4)	0.048 (4)	0.037 (4)	−0.011 (3)	0.007 (3)	−0.001 (3)
C13	0.050 (4)	0.064 (4)	0.038 (4)	−0.004 (3)	0.008 (3)	−0.004 (3)
C14	0.051 (4)	0.048 (4)	0.050 (4)	0.002 (3)	0.004 (3)	−0.009 (3)
C15	0.043 (3)	0.042 (3)	0.031 (3)	−0.007 (3)	−0.004 (2)	0.003 (3)
C16	0.043 (3)	0.046 (4)	0.058 (4)	−0.010 (3)	0.002 (3)	0.001 (3)
C17	0.044 (3)	0.041 (3)	0.046 (4)	−0.004 (3)	0.005 (3)	−0.008 (3)
C18	0.066 (4)	0.048 (4)	0.054 (4)	−0.008 (3)	−0.004 (3)	−0.008 (3)
C19	0.086 (5)	0.068 (5)	0.043 (4)	0.007 (4)	−0.012 (4)	−0.009 (4)
C20	0.116 (7)	0.052 (4)	0.041 (4)	−0.001 (5)	0.012 (4)	−0.002 (3)
C21	0.094 (6)	0.053 (4)	0.058 (5)	−0.016 (4)	0.001 (4)	0.007 (4)
C22	0.056 (4)	0.046 (4)	0.055 (4)	−0.008 (3)	0.006 (3)	−0.004 (3)
C23	0.056 (4)	0.093 (6)	0.049 (4)	−0.021 (4)	−0.005 (3)	0.012 (4)
C24	0.054 (4)	0.125 (8)	0.057 (5)	0.003 (5)	−0.007 (4)	0.010 (5)
C25	0.059 (5)	0.123 (7)	0.041 (4)	−0.019 (5)	−0.014 (4)	0.012 (4)
C26	0.056 (4)	0.060 (4)	0.028 (3)	−0.021 (3)	−0.003 (3)	0.010 (3)
C27	0.061 (4)	0.064 (4)	0.041 (4)	−0.006 (3)	−0.001 (3)	0.001 (3)
C28	0.040 (3)	0.052 (4)	0.038 (4)	0.000 (3)	0.000 (3)	−0.003 (3)
C29	0.048 (4)	0.058 (4)	0.048 (4)	−0.005 (3)	0.003 (3)	−0.006 (3)
C30	0.072 (5)	0.061 (5)	0.055 (5)	−0.001 (4)	0.015 (4)	−0.017 (4)
C31	0.068 (5)	0.049 (4)	0.068 (5)	−0.003 (4)	0.005 (4)	0.003 (4)
C32	0.054 (4)	0.066 (5)	0.037 (4)	0.003 (3)	0.005 (3)	0.003 (3)
C33	0.046 (3)	0.053 (4)	0.033 (3)	0.006 (3)	0.001 (3)	−0.006 (3)

### Geometric parameters (Å, º)

**Table:**

Ag1—N1	2.212 (4)	C7—C8	1.366 (10)
Ag1—N4	2.235 (4)	C7—H7	0.9300
Ag1—N7	2.660 (5)	C8—C9	1.388 (10)
Ag1—O4	2.691 (5)	C8—H8	0.9300
O1—C11	1.359 (8)	C9—C10	1.372 (10)
O1—H1O	0.839 (10)	C9—H9	0.9300
O2—C22	1.368 (8)	C10—C11	1.391 (9)
O2—H2O	0.839 (10)	C10—H10	0.9300
O3—C33	1.357 (7)	C12—C13	1.369 (8)
O3—H3O	0.840 (10)	C12—H12	0.9300
O4—N10	1.229 (7)	C13—C14	1.370 (9)
O5—N10	1.219 (7)	C13—H13	0.9300
O6—N10	1.215 (7)	C14—H14	0.9300
N1—C1	1.322 (7)	C16—C17	1.496 (9)
N1—C4	1.362 (7)	C16—H16A	0.9700
N2—C3	1.333 (8)	C16—H16B	0.9700
N2—C4	1.337 (7)	C17—C18	1.377 (9)
N3—C4	1.337 (7)	C17—C22	1.406 (8)
N3—C5	1.457 (8)	C18—C19	1.385 (10)
N3—H3N	0.880 (10)	C18—H18	0.9300
N4—C12	1.332 (7)	C19—C20	1.388 (10)
N4—C15	1.355 (7)	C19—H19	0.9300
N5—C14	1.326 (8)	C20—C21	1.371 (11)
N5—C15	1.345 (7)	C20—H20	0.9300
N6—C15	1.332 (7)	C21—C22	1.369 (10)
N6—C16	1.447 (7)	C21—H21	0.9300
N6—H6N	0.879 (10)	C23—C24	1.367 (10)
N7—C23	1.316 (9)	C23—H23	0.9300
N7—C26	1.349 (8)	C24—C25	1.365 (10)
N8—C25	1.332 (9)	C24—H24	0.9300
N8—C26	1.351 (8)	C25—H25	0.9300
N9—C26	1.341 (8)	C27—C28	1.514 (9)
N9—C27	1.454 (8)	C27—H27A	0.9700
N9—H9N	0.880 (10)	C27—H27B	0.9700
C1—C2	1.378 (9)	C28—C29	1.389 (8)
C1—H1A	0.9300	C28—C33	1.391 (8)
C2—C3	1.366 (9)	C29—C30	1.373 (9)
C2—H2A	0.9300	C29—H29	0.9300
C3—H3A	0.9300	C30—C31	1.373 (10)
C5—C6	1.513 (9)	C30—H30	0.9300
C5—H5A	0.9700	C31—C32	1.378 (9)
C5—H5B	0.9700	C31—H31	0.9300
C6—C7	1.376 (9)	C32—C33	1.388 (9)
C6—C11	1.400 (8)	C32—H32	0.9300
			
N1—Ag1—N4	174.16 (17)	N4—C12—H12	119.0
N1—Ag1—N7	95.84 (18)	C13—C12—H12	119.0
N4—Ag1—N7	85.61 (17)	C12—C13—C14	116.9 (6)
N1—Ag1—O4	90.50 (16)	C12—C13—H13	121.6
N4—Ag1—O4	95.16 (17)	C14—C13—H13	121.6
N7—Ag1—O4	90.16 (16)	N5—C14—C13	123.3 (6)
C11—O1—H1O	107 (6)	N5—C14—H14	118.3
C22—O2—H2O	112 (6)	C13—C14—H14	118.3
C33—O3—H3O	109 (6)	N6—C15—N5	119.3 (5)
N10—O4—Ag1	135.7 (4)	N6—C15—N4	116.5 (5)
C1—N1—C4	116.4 (5)	N5—C15—N4	124.2 (5)
C1—N1—Ag1	115.5 (4)	N6—C16—C17	114.9 (5)
C4—N1—Ag1	124.1 (4)	N6—C16—H16A	108.6
C3—N2—C4	117.4 (5)	C17—C16—H16A	108.6
C4—N3—C5	123.6 (5)	N6—C16—H16B	108.6
C4—N3—H3N	122 (4)	C17—C16—H16B	108.6
C5—N3—H3N	113 (4)	H16A—C16—H16B	107.5
C12—N4—C15	117.1 (5)	C18—C17—C22	118.0 (6)
C12—N4—Ag1	119.2 (4)	C18—C17—C16	124.4 (5)
C15—N4—Ag1	123.0 (4)	C22—C17—C16	117.7 (6)
C14—N5—C15	116.4 (5)	C17—C18—C19	121.7 (6)
C15—N6—C16	126.0 (5)	C17—C18—H18	119.2
C15—N6—H6N	119 (4)	C19—C18—H18	119.2
C16—N6—H6N	114 (4)	C18—C19—C20	118.6 (7)
C23—N7—C26	116.5 (6)	C18—C19—H19	120.7
C23—N7—Ag1	109.1 (4)	C20—C19—H19	120.7
C26—N7—Ag1	125.0 (5)	C21—C20—C19	121.1 (7)
C25—N8—C26	115.9 (6)	C21—C20—H20	119.4
C26—N9—C27	123.6 (5)	C19—C20—H20	119.4
C26—N9—H9N	110 (4)	C22—C21—C20	119.6 (7)
C27—N9—H9N	122 (4)	C22—C21—H21	120.2
O6—N10—O5	122.5 (7)	C20—C21—H21	120.2
O6—N10—O4	118.0 (6)	O2—C22—C21	123.5 (6)
O5—N10—O4	119.4 (7)	O2—C22—C17	115.4 (6)
N1—C1—C2	123.4 (6)	C21—C22—C17	121.0 (7)
N1—C1—H1A	118.3	N7—C23—C24	123.8 (7)
C2—C1—H1A	118.3	N7—C23—H23	118.1
C3—C2—C1	116.0 (6)	C24—C23—H23	118.1
C3—C2—H2A	122.0	C25—C24—C23	115.5 (8)
C1—C2—H2A	122.0	C25—C24—H24	122.2
N2—C3—C2	122.8 (6)	C23—C24—H24	122.2
N2—C3—H3A	118.6	N8—C25—C24	123.7 (7)
C2—C3—H3A	118.6	N8—C25—H25	118.2
N3—C4—N2	119.0 (5)	C24—C25—H25	118.2
N3—C4—N1	117.3 (5)	N9—C26—N7	117.7 (6)
N2—C4—N1	123.6 (5)	N9—C26—N8	118.1 (6)
N3—C5—C6	114.3 (6)	N7—C26—N8	124.2 (6)
N3—C5—H5A	108.7	N9—C27—C28	114.5 (5)
C6—C5—H5A	108.7	N9—C27—H27A	108.6
N3—C5—H5B	108.7	C28—C27—H27A	108.6
C6—C5—H5B	108.7	N9—C27—H27B	108.6
H5A—C5—H5B	107.6	C28—C27—H27B	108.6
C7—C6—C11	117.5 (6)	H27A—C27—H27B	107.6
C7—C6—C5	124.1 (5)	C29—C28—C33	118.0 (6)
C11—C6—C5	118.4 (6)	C29—C28—C27	123.3 (6)
C8—C7—C6	121.9 (6)	C33—C28—C27	118.6 (5)
C8—C7—H7	119.0	C30—C29—C28	121.0 (6)
C6—C7—H7	119.0	C30—C29—H29	119.5
C7—C8—C9	120.4 (7)	C28—C29—H29	119.5
C7—C8—H8	119.8	C29—C30—C31	120.6 (6)
C9—C8—H8	119.8	C29—C30—H30	119.7
C10—C9—C8	119.2 (7)	C31—C30—H30	119.7
C10—C9—H9	120.4	C30—C31—C32	119.6 (7)
C8—C9—H9	120.4	C30—C31—H31	120.2
C9—C10—C11	120.0 (6)	C32—C31—H31	120.2
C9—C10—H10	120.0	C31—C32—C33	120.1 (6)
C11—C10—H10	120.0	C31—C32—H32	119.9
O1—C11—C10	122.8 (6)	C33—C32—H32	119.9
O1—C11—C6	116.3 (6)	O3—C33—C32	122.8 (5)
C10—C11—C6	120.9 (6)	O3—C33—C28	116.6 (6)
N4—C12—C13	122.1 (6)	C32—C33—C28	120.6 (6)
			
N1—Ag1—O4—N10	−128.7 (6)	C12—C13—C14—N5	−0.1 (10)
N4—Ag1—O4—N10	52.8 (6)	C16—N6—C15—N5	5.0 (10)
N7—Ag1—O4—N10	−32.9 (6)	C16—N6—C15—N4	−175.2 (6)
N7—Ag1—N1—C1	122.7 (4)	C14—N5—C15—N6	179.8 (6)
O4—Ag1—N1—C1	−147.1 (4)	C14—N5—C15—N4	0.1 (9)
N7—Ag1—N1—C4	−34.0 (5)	C12—N4—C15—N6	179.3 (5)
O4—Ag1—N1—C4	56.2 (4)	Ag1—N4—C15—N6	−10.6 (7)
N7—Ag1—N4—C12	108.4 (4)	C12—N4—C15—N5	−1.0 (8)
O4—Ag1—N4—C12	18.7 (5)	Ag1—N4—C15—N5	169.1 (4)
N7—Ag1—N4—C15	−61.5 (4)	C15—N6—C16—C17	−84.9 (8)
O4—Ag1—N4—C15	−151.3 (4)	N6—C16—C17—C18	−1.4 (9)
N1—Ag1—N7—C23	132.8 (5)	N6—C16—C17—C22	178.5 (5)
N4—Ag1—N7—C23	−52.8 (5)	C22—C17—C18—C19	−1.3 (9)
O4—Ag1—N7—C23	42.3 (5)	C16—C17—C18—C19	178.5 (6)
N1—Ag1—N7—C26	−12.2 (5)	C17—C18—C19—C20	1.2 (10)
N4—Ag1—N7—C26	162.2 (5)	C18—C19—C20—C21	−1.8 (11)
O4—Ag1—N7—C26	−102.7 (5)	C19—C20—C21—C22	2.4 (11)
Ag1—O4—N10—O6	77.5 (8)	C20—C21—C22—O2	178.3 (7)
Ag1—O4—N10—O5	−106.3 (7)	C20—C21—C22—C17	−2.5 (11)
C4—N1—C1—C2	2.2 (9)	C18—C17—C22—O2	−178.8 (6)
Ag1—N1—C1—C2	−156.3 (5)	C16—C17—C22—O2	1.3 (8)
N1—C1—C2—C3	2.7 (10)	C18—C17—C22—C21	2.0 (9)
C4—N2—C3—C2	−0.2 (10)	C16—C17—C22—C21	−177.9 (6)
C1—C2—C3—N2	−3.7 (10)	C26—N7—C23—C24	0.0 (11)
C5—N3—C4—N2	0.7 (9)	Ag1—N7—C23—C24	−148.3 (7)
C5—N3—C4—N1	179.8 (6)	N7—C23—C24—C25	5.0 (12)
C3—N2—C4—N3	−175.2 (6)	C26—N8—C25—C24	0.5 (12)
C3—N2—C4—N1	5.8 (9)	C23—C24—C25—N8	−5.2 (13)
C1—N1—C4—N3	174.3 (5)	C27—N9—C26—N7	−170.8 (6)
Ag1—N1—C4—N3	−29.2 (7)	C27—N9—C26—N8	9.7 (9)
C1—N1—C4—N2	−6.7 (9)	C23—N7—C26—N9	175.1 (6)
Ag1—N1—C4—N2	149.8 (5)	Ag1—N7—C26—N9	−42.1 (7)
C4—N3—C5—C6	74.2 (8)	C23—N7—C26—N8	−5.5 (9)
N3—C5—C6—C7	4.3 (9)	Ag1—N7—C26—N8	137.3 (5)
N3—C5—C6—C11	−176.3 (5)	C25—N8—C26—N9	−175.4 (6)
C11—C6—C7—C8	−0.9 (9)	C25—N8—C26—N7	5.2 (10)
C5—C6—C7—C8	178.6 (6)	C26—N9—C27—C28	68.8 (8)
C6—C7—C8—C9	0.4 (10)	N9—C27—C28—C29	7.2 (9)
C7—C8—C9—C10	0.2 (11)	N9—C27—C28—C33	−172.4 (5)
C8—C9—C10—C11	−0.2 (11)	C33—C28—C29—C30	−3.0 (9)
C9—C10—C11—O1	179.4 (6)	C27—C28—C29—C30	177.4 (6)
C9—C10—C11—C6	−0.4 (10)	C28—C29—C30—C31	2.1 (11)
C7—C6—C11—O1	−178.9 (6)	C29—C30—C31—C32	−1.2 (11)
C5—C6—C11—O1	1.7 (8)	C30—C31—C32—C33	1.3 (10)
C7—C6—C11—C10	0.9 (9)	C31—C32—C33—O3	179.3 (6)
C5—C6—C11—C10	−178.6 (6)	C31—C32—C33—C28	−2.3 (10)
C15—N4—C12—C13	1.4 (9)	C29—C28—C33—O3	−178.4 (5)
Ag1—N4—C12—C13	−169.1 (5)	C27—C28—C33—O3	1.2 (8)
N4—C12—C13—C14	−0.9 (10)	C29—C28—C33—C32	3.1 (9)
C15—N5—C14—C13	0.5 (10)	C27—C28—C33—C32	−177.3 (6)

### Hydrogen-bond geometry (Å, º)

**Table:**

*D*—H···*A*	*D*—H	H···*A*	*D*···*A*	*D*—H···*A*
O1—H1o···N8^i^	0.84 (1)	2.00 (3)	2.800 (7)	158 (9)
O2—H2o···N5^ii^	0.84 (1)	1.95 (1)	2.788 (6)	177 (9)
O3—H3o···N2^iii^	0.84 (1)	1.99 (2)	2.818 (6)	171 (8)
N3—H3n···O4	0.88 (1)	2.22 (2)	3.073 (7)	164 (6)
N6—H6n···O5^iv^	0.88 (1)	2.09 (4)	2.879 (8)	149 (6)
N9—H9n···O6^iv^	0.88 (1)	2.28 (4)	3.033 (8)	143 (6)

Symmetry codes: (i) −*x*+1, −*y*+1, −*z*; (ii) −*x*+2, −*y*+2, −*z*+1; (iii) −*x*+2, −*y*+1, −*z*; (iv) *x*+1, *y*, *z*.
